# An unusual case of T-cell acute lymphoblastic leukemia in a patient with BCR-ABL positive chronic myeloid leukemia and Gaucher disease

**DOI:** 10.1016/j.amsu.2021.102565

**Published:** 2021-07-14

**Authors:** Hamdi Al-Janazreh, Yousef S. Abuzneid, Iman Khamayseh, Fortunato Morabito, Bilal Alqam, Rosaline M.F. Abusabbah, Fatima K. Mustafa, Shifa Sarahneh

**Affiliations:** a Al-Quds University Faculty of Medicine, Jerusalem, Palestine; b Hematology Department and Bone Marrow Transplant Unit, Cancer Care Center, Augusta Victoria Hospital, Jerusalem, Palestine

**Keywords:** CML, T-ALL, Blast crisis, Transformation, Case report

## Abstract

**Background:**

Chronic myelogenous leukemia (CML) is a chronic myeloproliferative disease characterized by a massive overproduction of myeloid cells. It is associated with the Philadelphia chromosome [Ph1, t (9; 22) (q34; q11)] or BCR-ABL fusion gene. CML usually undergoes a triphasic clinical course ending in a blast crisis, an accelerated phase of blasts and promyelocyte production. Ten percent of CML patients reach the blast crisis phase, with 20–30% of leukemias belonging to B-cell lymphoid lineage. However, a transformation of CML into T-cell acute lymphoblastic leukemia (T-ALL) is rare.

**Case presentation:**

We present a 56-year-old male patient, known case of hypertension and Ph1 CML of eight years with a family history of Gaucher disease who developed T-ALL. The patient presented with lymphadenopathy and severe anemia, needing packed RBC transfusion, neutropenia and thrombocytopenia at the admission. However, the monocytes and basophils percentage were high. The patient underwent a cervical lymph node core biopsy, and the immunohistochemistry stains showed an invasion of neoplastic cells positive for CD3, CD5, BCL2, CD34, TdT and focally positive for C-Kit and negative for CD20, CD56 and pan-CK. These histopathology features were consistent with T-cell acute lymphoblastic leukemia (T-ALL).

**Conclusion:**

Blast crisis remain a challenge in CML management. It's of great importance to do a full proper workup including lymph nodes biopsies. The aim is to reverse blast crisis and restore the chronic phase.

## Highlights

• Even if CML normally transforms into B-ALL, it can also manifest as T-ALL.
• Careful history taking, meticulous physical examination, and investigations are necessary.
• Avoiding the blast crisis is very important in order to prevent its transformation.

## 1 Introduction

Chronic myelomonocytic leukemia (CML) is a myeloproliferative neoplasm originating from the pluripotent bone marrow stem cell whose hallmark is represented by ABL1 gene translocation from chromosome 9 to the region of the BCR gene on chromosome 22 (BCR-ABL1). BCR-ABL1 fusion gene encodes an abnormal protein that activates tyrosine kinase which is responsible for the activation of signal transduction pathways, causing the abnormal bone marrow proliferation and the clinical and morphologic manifestations of this type of leukemia [1].

The annual incidence of this condition is 1.0–1.5 per 100,000 persons. In the Western world, the median age of onset is 50–60 years, which reflects the average age of the population. 50% of the patients are asymptomatic and are diagnosed after a routine blood test checkup for unrelated reasons; however, in the developed countries, patients may be symptomatic and experience lethargy, weight loss, unusual bleeding, diaphoresis, anemia and splenomegaly [4].

It is well known that CML can have three different phases [2,3].

• First stage (chronic phase): cancer grows very slowly, and the accumulation of myeloid cells characterizes the hematological picture. This phase can last for years.
• Second stage (accelerated phase): cancer cells grow faster.
• The last stage (blast phase): cancer is fast-growing and can be fatal. Most of the patients who progressed to blast crisis develop AML and others B cell ALL. However, progression to T cell ALL has been rarely seen.

The prognosis of CML was improved according to a study published in 2015 saying that the 5-year survival rate was more than doubled, from 31% in the early 1990 to 69.2% for patients diagnosed from 2009 to 2015.

New targeted therapies, aimed to block the constitutively active tyrosine-kinase BCR-ABL1 oncogene, changed the disease's natural history. For this reason, tyrosine-kinase inhibitors as the first-line therapy offer to many patients a life expectancy equivalent to healthy people. However, the median survival of CML patients developing blast crisis is not improved notably [25].

According to the WHO classification of acute lymphoblastic leukemia (ALL), 70–75% of the adults are precursors of B-ALL. T-cell acute lymphoblastic leukemia/lymphoblastic lymphoma are relatively uncommon (25%) in adults but commoner than pediatric T-ALL (10%). Only about 25 cases of de novo Ph + T cell ALL and 44 cases of Ph + T-ALL in a blastic phase of CML have been reported [5].

Few cases of Philadelphia positivity in T-ALL are reported in the literature. Hence, the clinical relevance and prognostic significance of this entity is yet unknown. T-cell lymphoid blast crises in CML is always a close differential diagnosis with Ph + T-cell ALL [[6], [7], [8], [9], [10]].

The presence of CML history (long duration of symptoms, old age, massive splenomegaly, previous diagnosis of CML), increasing number of residual circulating granulocytic precursors and major BCR-ABL breakpoint transcript favors a T-cell lymphoblastic crisis of CML. However, younger age, bone marrow involvement by numerous blasts and presence of minor BCR-ABL transcript favors Ph positive pre-T-ALL [5].

The work has been reported in line with the SCARE 2020 criteria [27].

## 2 Case presentation

A 56-year-old male patient, a known case of hypertension, a positive family history of Gaucher disease and Ph1-positive CML on Imatinib as treatment for eight years, came to our hospital in October 2020 with a clinical picture characterized by pancytopenia and lymphadenopathy.

The patient underwent work up CBC that showed a low level of hemoglobin essentially requiring blood transfusion to stabilize his status.

Another CBC post-transfusion was executed, showing a remaining low hemoglobin level (9.89g/dL), low PLT count (111 × 10^3^/μL), with relatively average neutrophil percentage (31.6%). In contrast, the monocyte (23.3%) and the basophil (7.56%) percentages were increased.

Also, electrolytes were imbalanced with a slight reduction in sodium (135 mmol/L) and chloride (91.6 mmol/L).

HIV, HCV and HVB tests were negative while antibodies against hepatitis Bs antigen resulted positive.

The microbiological analysis of urine was substantially negative, and a surveillance nasal swab culture showed no MRSA, CRE or VRE growth. Finally, PCR from peripheral blood cells documented EBV and CMV viral overload.

After a confirmatory real-time PCR for major BCR-ABL fusion transcript was performed and detected from a peripheral blood sample, the patient started therapy with a second-generation TK inhibitor (Nilotinib) and an extensive molecular workup continued to survey the status.

A whole-body CT-scan showed normal ventricular system and basal cisterns, no intracranial hemorrhage, no enhancing lesions and clear paranasal sinuses. A conglomerate of enlarged pathological lymph nodes, some with necrotic centers, involving the whole right cervical region and mainly along the jugular levels (measuring 6.5 × 5 cm in maximum axial measurement), exerting mass effects upon the jugular vein but with a patent carotid artery was also documented at the neck. There were also multiple scattered left cervical enlarged lymph nodes measuring up to 1.5 cm.

**Fig. 1:**
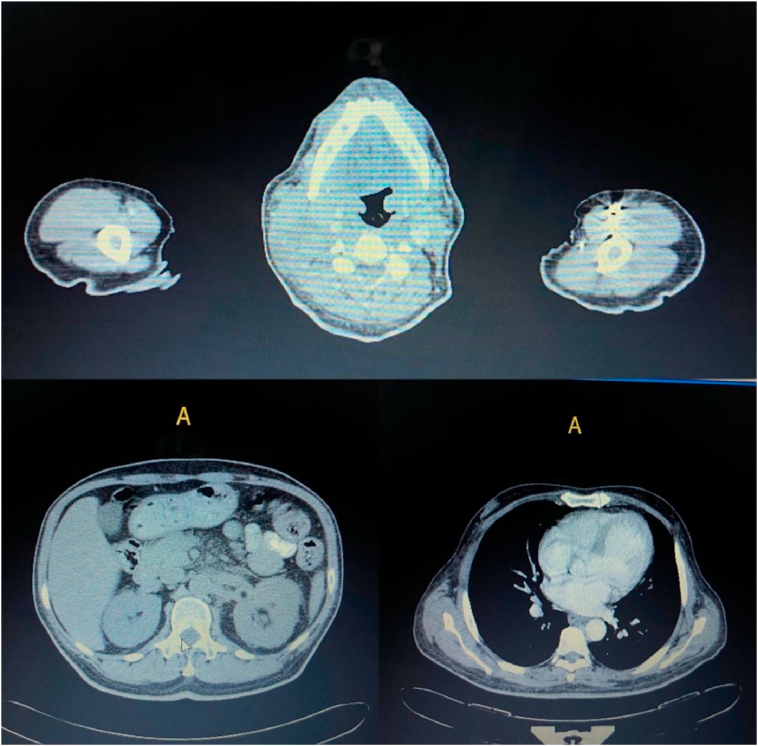
CT scan showing a conglomerate of enlarged pathological lymph nodes, some with necrotic centers, in the right cervical region, right and left side of the chest and peri-aortic region.

Bilateral axial pathological lymph nodes (the largest ones were 2 × 1.3 cm on the right side and 2.1 × 1.3 cm on the left side) were detected at the chest level. However, both lungs were clear, without focal lesions, no pathological mediastinal or hilar lymphadenopathy and normal heart and great vessels. The liver was homogenous and without focal lesions or intrahepatic biliary tree dilation; the spleen, pancreas, adrenals, gallbladder and kidneys were normal; and there was no intra-abdominal or pelvic free fluid collection. However, there were several enlarged retro-peritoneal and inguinal lymph nodes mainly at the upper peri-aortic region, measuring up to 1.8 × 1.2 cm (Fig. 1).

The patient also underwent an ultrasound-guided core biopsy of the right cervical lymph node. The histopathological evaluation proved features consistent with T-ALL and immunohistochemistry stains showed neoplastic cells’ positivity for CD3, CD5, BCL2, CD34, TdT and focally positive for C-Kit but they were negative for CD20, CD56 and pan-CK. Ki-67 was positive in about 90% of the neoplastic cells too.

Steroids were added to the current therapy with Nilotinib.

**Fig. 2:**
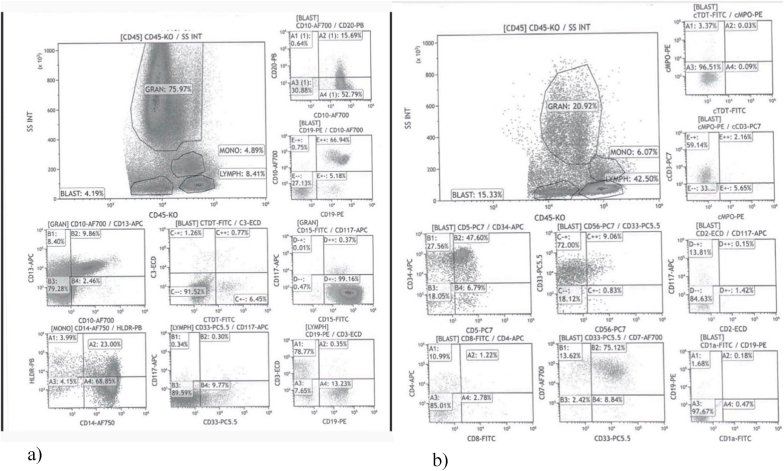
a) Flow cytometry at the beginning of the case. b) Flow cytometry from April 2021.

After twelve days, we performed a bone marrow trephine biopsy which showed some reactive lymphoid follicles, a TdT and CD34 increased mildly in blasts up to 10% and CD3 and CD20 stained reactive lymphocytes. We also asked for a flow cytometry analysis which showed involvement by T- ALL with positive cells in the blast region (positive for CD5 47%; CD34 75%; CD33 83%; CD7 88%; and DIM positive for cytoplasmic CD3); 15.5% blasts; 6% monocytes; 43% lymphocytes; and 21% granulocytes (Fig. 2).

**Fig. 3:**
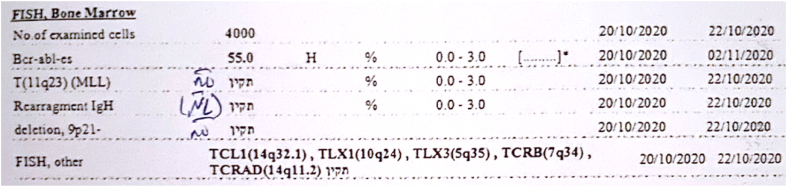
FISH test showing mutation such as TCL1 (14q32.1), TLX1 (10q24), TLX3 (5q35), TCRB(7q34), TCRAD (14q11.2).

FISH/CISH and the karyotype results came back to us. The FISH results showed that the T (11q23) (MLL), the rearrangement IgH and the deletion for 9p21 were normal; however, there were present other mutation such as TCL1 (14q32.1), TLX1 (10q24), TLX3 (5q35), TCRB(7q34), TCRAD (14q11.2). (Fig. 3).

**Fig. 4:**
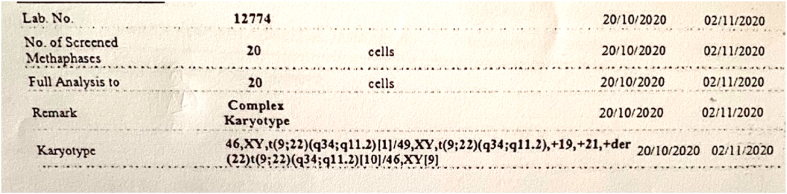
Karyotype showing 46,XY, t (9; 22) (q34; q11.2) [1]/49,XY, t (9; 22) (q34; q11.2), +19, +21, +der (22)t (9; 22) (q34; q11.2) [10]/46, XY [9].

The karyotype showed 46,XY, t (9; 22) (q34; q11.2) [1]/49,XY, t (9; 22) (q34; q11.2), +19, +21, +der (22)t (9; 22) (q34; q11.2) [10]/46, XY [9] (Fig. 4).

We also did a TP53 mutation analysis, but no pathogenic variant was detected.

Furthermore, a cerebrospinal fluid (CSF) analysis was elaborated, showing an increase in CSF protein (46.7 when the normal range is 15–45 mg/dL) but otherwise normal. A CSF cytology showed no blasts involvement.

CBC showed an improvement in WBCs count (2.25 × 10^3^/μL), hemoglobin level (10.3 g/dL) and PLTs count (145 × 10^3^/μL).

**Fig. 5:**
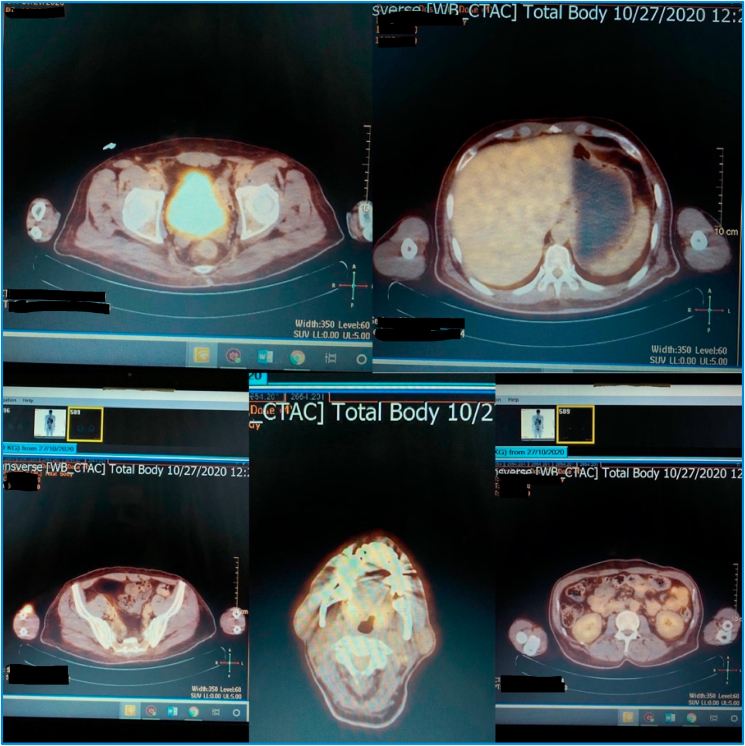
PET CT-scan showing hypermetabolic sub-centimetric cervical lymph nodes in the right side, the mediastinum, para-spinal region and abdominal and pelvic regions.

Few days later, the patient underwent a further whole-body PET-CT scan that showed mild hypermetabolic sub-centimetric cervical lymph nodes in the neck's right side (SUV max up to 2.4). Moreover, physiologic myocardial FDG metabolic activity with no evidence of hypermetabolic mediastinal or hilar lymphadenopathy or pulmonary nodules. There were two peripheral sub-pleural mildly hypermetabolic nodules in the right lung, the most prominent in the para-spinal region with SUV max up to 3.9. A physiologic limit of FDG metabolic activity in the liver (SUV max: 3.5) as well as the spleen and bowel with no evidence of active focal lesions were detected on the abdomen and pelvis. There was no evidence of hypermetabolic abdominal or pelvic lymph nodes. The musculoskeletal apparatus showed a limit of FDG metabolic activity in the musculoskeletal system with no evidence of active focal lesions (Fig. 5).

A real-time follow-up PCR for major BCR-ABL still detected the fusion transcript.

Repeated CSF analysis showed higher CSF glucose (98.4 when the normal is 40–70 mg/dL); however, CSF protein was normal, and the CSF cytology excluded the presence of blasts.

Finally, the patient then underwent two cycles of Hyper-CVAD arm A + B to get complete remission as bridging to allogenic bone marrow transplant (BMT) and Nilotinib was continued (400 mg twice a day).

According to the flow cytometry performed in April 2021, there was no evidence of increased blast population in the bone marrow and showed 4.2% cells in the blast region; 8.4% lymphocytes; 5% monocytes; and 76% granulocytes (Fig. 2).

## 3 Discussion

CML is clinically classified into three phases: chronic phase (CP), accelerated phase (AP), and blast phase (BP), with 90–95% of patients presenting in the CP [11]. Acute transformation into BP occurs in 76% of patients. BP may be myeloid (70%) or lymphoid (30%). Of the lymphoblastic phenotype, most will exhibit the B-cell lineage, whereas T-lymphoblastic BP of CML has been rarely reported [12,13].

The mean time interval between the diagnosis of CML and acute blast phase diagnosis is 3.5 years with a range of 1–10 years. The prognosis of CML in the blast phase is poor, with a median survival of 6 months for myeloid type and 12 months for lymphoid type [12,14].

Notably, the introduction of the first, second, and even third generation of TK inhibitors undoubtedly radically changed the overall CML prognosis. However, some specific points deserve to be discussed in our case.

First, the advent of generic imatinib, now available worldwide, significantly improved the cost-effectiveness of initial treatment of chronic phase CML. Moreover, since there is no significant survival advantage and it has a trivial cumulative long-term toxicity, the second generation TKIs as first-line therapy has been reconsidered. The cumulative incidence of BC, in the era of more effective treatment, dramatically changed from about 70% at 8 years to the current 5% during imatinib therapy [26].

The above consideration is to keep the treatment received by our patient. Unfortunately, no information on the molecular monitoring of treatment with imatinib was available for our patient. BC is the consequence of the continued exposure of cells to BCR-ABL activity determining genomic instability by more mutations and other genetic aberrations [4].

In this respect, the molecular monitoring of treatment, almost exclusively performed by quantitative PCR, is mandatory to guarantee optimal clinical results. Early therapeutic switch from the first to the second generation TKIs was systematically undertaken and represent a milestone of the modern therapeutic strategy in CML [25].

The most consistent cause of therapy resistance is represented by the development of resistant mutations in advancing CP and its association with higher levels of BCR-ABL gene, mostly anticipated by the constant overtime increment of the fusion gene [15]. Regrettably, neither BCR-ABL1 gene mutation nor mutations most frequently observed involving p53 (in 25% of myeloid BC) and p16/AKT (in about 15% of lymphoid BC) have been tested in our patient. Complex chromosomal aberrations, among other karyotype alterations, are also frequently observed (trisomy 8, isochromosome 17, trisomy 19) [17]. Lymphoid cells in BC generally express terminal deoxynucleotidyl transferase (TdT) [18], as exhibited in our patient.

The reported clinical features in T-cell blast crisis cases include B symptoms and lymphadenopathy; however, many patients are diagnosed due to abnormal CBC results [19,20]. Most BC patients have a hematological picture of hemoglobin less than 10 g/dL, white blood cell count more than 10 × 10^9^, and platelet count less than 100 × 10^9^ [12]. Noteworthy, it is essential to biopsy both the bone marrow and lymph nodes, as the lymph nodes may be the initial site of blast transformation [20]. Our patient presented with the clinical picture of pancytopenia and lymphadenopathy; however, involvement of other extramedullary sites as liver, spleen, and mediastinum were not identified.

The WHO's proposed diagnostic criteria for CML, BC requires one or more of following: blasts 20% or more of peripheral blood leukocytes or nucleated bone marrow cells, extramedullary blast proliferation, large foci or clusters of blasts in bone marrow biopsy [16].

Treatment options for T-cell lymphoblastic crises of CML in the BC phase include ALL chemotherapy regimens mainly associated with different TKI such as imatinib [21,22]. Complete molecular and cytogenetic response with this regimen has been reported [23]. Allogeneic stem cell transplantation represents the only curative treatment option. Nevertheless, the survival after allografted for BC CML is strongly associated with pretransplant remission status. Accordingly, BC status at transplant is the most potent negative prognostic factor for overall survival [24].

## 4 Conclusion

BC remains the major concern in the management of CML. This condition presents mainly with B symptoms and lymphadenopathy and blood work up abnormalities may also be an indicator. We should highlight the importance of the biopsy, especially of the lymph nodes, as they can be the initial site of blast transformation.

The prognosis and treatment vary according to the transformation in BC. Therefore, it's the physician's responsibility to properly evaluate all patients with CML and consider its rare transformation to T-ALL which needs aggressive treatment.

The goal is to reverse BC and restore the chronic phase, hopefully with a cytogenic and/or molecular remission. The main form of therapy should be a TKI followed rapidly by allo-SCT. TKI with leukemia-type induction therapy, such as vincristine plus prednisone or Hyper-CVAD combined with TKI in lymphoid BC, is advisable.

Allogeneic stem cell transplantation; represents the only curative treatment option in patients with BC. However, this procedure is challenging and demanding in Palestine due to the inadequate facilities and low income.

We hope that reporting our patient's presentation will add more information about this rare transformation as its prognostic significance is still poorly understood, especially in a developing country.

## Authors’ contributions

Study concept or design: Hamdi Al-Janazreh.

Writing the manuscript: Yousef S. Abuzneid, Iman Khamayseh, Fortunato Morabito, Bilal Alqam, Rosaline M. F. Abusabbah, Fatima K. Mustafa and Shifa Sarahneh.

Review & editing the manuscript: Yousef S. Abuzneid, Hamdi Al-Janazreh.

## Please state any conflicts of interest

There is no conflict of interest.

## Please state any sources of funding for your research

No funding or grant support.

## Conflicts of interest

There is no conflict of interest.

## Sources of funding

This research did not receive any specific grant from funding agencies in the public, commercial, or not-for-profit sectors.

## Ethical approval

The study is exempt from ethical approval in our institution.

## Consent

Written informed consent was obtained from the patient's parents for publication of this case report and accompanying images since the patient is a minor. A copy of the written consent is available for review by the Editor-in-Chief of this journal on request.

## Guarantor

Yousef S. Abuzneid.

## Ethical approval

The study is exempt from ethical approval in our institution.

## Sources of funding

No funding or grant support.

## Registration of research studies

Not applicable.

## Declaration of competing interest

There is no conflict of interest.
